# The uncanny drawings of a 10-year-old Portuguese boy

**DOI:** 10.1017/S2045796020000177

**Published:** 2020-02-26

**Authors:** J. P. Fróis

**Affiliations:** Center for Phenomenological Psychology and Aesthetics, CPPA, University of Copenhagen, Denmark

**Keywords:** Art, art brut, child psychiatry, psychosis

*The child's drawing is not an image but a sign, and as a sign it is situated in the pictographic domain and not in the representative one: in the domain of writing and not in that of painting*.

Cesare Brandi, 1960

Art works created by the mentally ill spring from the same basic psychological roots as do the works of other artists.

Rudolf Arnheim, 1986

The science of the late 19th century profoundly influenced the way we understand children and the various dimensions of their intellectual and emotional development. One of these dimensions includes children's artistic creative urge as manifested in children's art productions. Child art has fascinated artists and scientists of all time. The English naturalist Charles Darwin included the analysis of his children's graphic productions in his studies. James Sully, in the context of the ‘Child Study Movement’, understood children's drawings as part of an evolutionary process developed step by step, by ‘discrete phases’. It was up to Italian archaeologist and art historian Corrado Ricci to publish the first full treatise on children's drawings, named *L'Arte dei Bambini* (1887), although earlier studies by Töpffer (1848) and Ruskin (1857) had alluded to the topic. The early years of the 20th century were decisive in generating artistic, anthropological, psychological and pedagogical knowledge of child art: members of all those scientific areas have established multiple correlations between children's drawings, the productions of remote tribes and those of the mentally ill (Boas, 1966; Peiry, 2016). Several collections of children's drawings and paintings became available and inspired such well-known artists as Jean Dubuffet, Paul Klee and Gabriele Münter. However, studies of drawings made by children with special educational needs are still relatively rare as compared to those made by other children (Selfe, 1977; Fineberg, 1997).

In the archives of the Medical School of the University of Lisbon there is a unique collection of crayon and watercolour drawings made by children of different ages who were being observed at the Pedagogical Medical Institute in Lisbon in the forties and fifties of the 20th century. According to the director of this Institute, Vítor Fontes (1893–1979), a prominent anatomist and psychiatrist, the medical-pedagogical approach had to be ‘the application of medical knowledge to the treatment of the individual with psychic or organic disability; a treatment which involves, in addition to other therapeutic means and in a more essential way, the pedagogical ones’ (Fontes, 1939). For the Institute's doctors, the patient's drawings helped to clarify the diagnosis of psychiatric disorders, whether in children or adults. The collection of 200 works by Fontes comes from a set of 30.000 existing works at the Institute and was for the first time shown at the ‘Scientific Exhibition of the 10th World Congress of Pediatrics’ (held in Lisbon, 1962). From this set of drawings, one drawing connected with a detailed description resulting from the clinical observation of a 10-year-old boy, specifically draws our attention. The original of the drawing (Fig. 1) is part of the collection mentioned before and the other one (Fig. 2) was reproduced in the article ‘A Case of Very Early Dementia’ published in the magazine *The Portuguese Child* (1942), along with 30 other drawings. The author of the drawings ‘rarely looked at people when he spoke, usually bowed his head, covered his ears with his hands and only then responded. In a nutshell, strange attitudes were noticed from the age of nine and a half years. A measles attack was followed by a major inflammation of the genitals (…) During the examination there were various stereotyped movements, sometimes with the upper limbs, sometimes shaking the body or the head’ (Fontes, 1942, p. 165, p. 166). The boy attended school from 7 to 9 years with moderate results. In his article, Fontes wrote that this was a case of the ‘schizophrenic syndrome’ and discussed the concept of ‘early dementia’ relying on the contributions of the Italian psychiatrist Sante De Sanctis (1862–1935) to the then emerging field of child psychiatry. For De Sanctis (1925) this type of dementia was a kind of ‘regression of the mental faculties’.

**Fig. 1.**
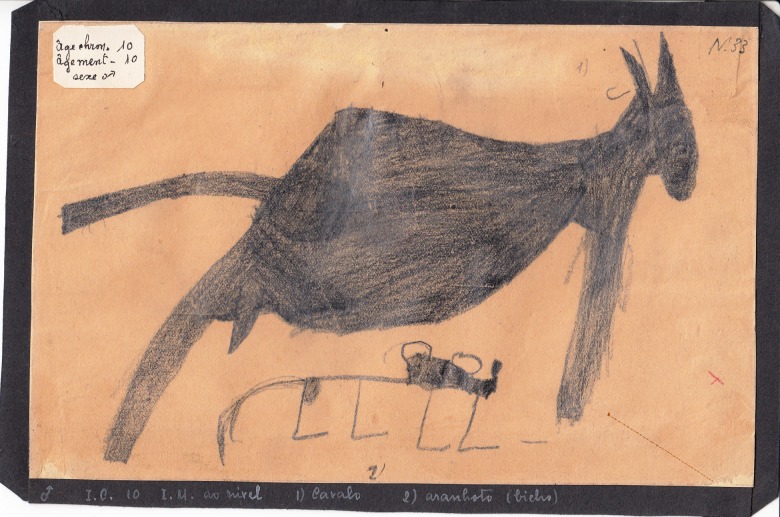
Zoomorphic drawings. Donkey and small beast, July 1942, Graphite pencil, 22 cm × 14 cm. Collection of the Institute of Anatomy, Lisbon School of Medicine, University of Lisbon.

**Fig. 2.**
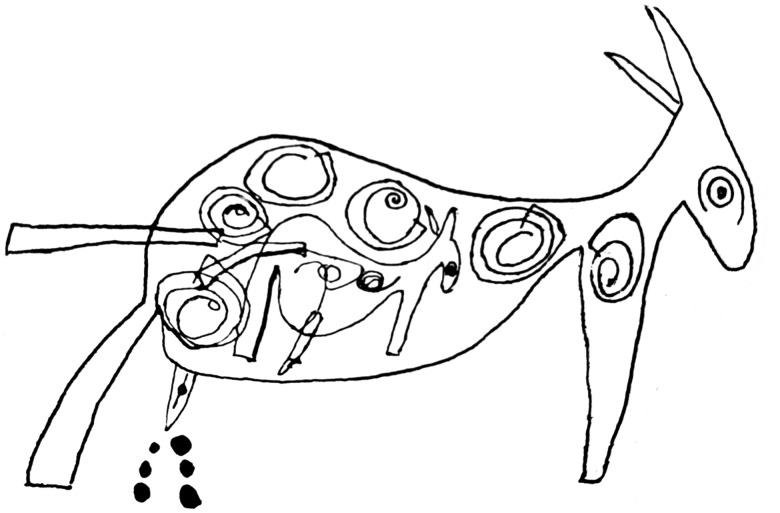
Zoomorphic drawing. Donkey, Graphite pencil, 22 cm × 14 cm, In Vítor Fontes, Um caso de demência precocíssima, A Criança Portuguesa, Boletim do Instituto Aurélio da Costa Ferreira, 1, 1942, p.175.

During his month-long stay at the Institute, the boy made 100 free drawings. All of the drawings that I have seen seem to fall outside the range of the standard drawings typical of children of that age, both what regards their meaning and their form. On the first day of his stay the boy drew an awkward picture of a man with arms and legs sticking out of a trunk (Fontes, 1942). Fontes classified the set of drawings into two broad categories: in the first category he included anthropomorphic drawings with a ‘vesanic’ appearance. They were like the drawings made by the ‘asylum freaks’, with explicit sexual signs, i.e. graphically strange human figures where the schematic character stands out (the boy referred to them as ‘skeletons’) and in the second category he included zoomorphic drawings that also have sexual content.

The two drawings shown here emerge with a well-defined contour. According to their author, they are ‘donkeys’ and as such they belong to the second category of drawings called zoomorphic by Fontes. In both of them the genital organ is patent and in one drawing the genital organ produces some sort of balls or drops reminding the act of urinating. In the drawing on the left there are two figures filled with graffiti of which the smallest according to the boy is a ‘small beast’. The drawing has a caption with the boy's chronological and mental age. In the drawing on the right (Fig. 2), with a precise contour, transparency as representation technique is deliberately used. Inside the larger figure (body) where a well-defined eye scheme appears, we find another figure that repeats the larger one. The small inner figure is surrounded by circular (spiraled) elements. By their graphic appearance the two drawings remind us of the (prehistoric) representations found in caves and they reveal the child's exceptional capacity to depict the total object within a peculiar syncretic vision. As a result, they strike us as truly mysterious.

## About the author

João Pedro Fróis is a University Professor and Researcher. His main interests lie in the Psychology of Visual Arts, Empirical Aesthetics and the Philosophy and History of Art Education. He has published articles in several journals on the Psychology of the Visual Arts. He has worked as a Rehabilitation Psychologist for more than a decade. As from 2014, he has been a Fellow of the International Association of Empirical Aesthetics. Research Associate of the Center for Phenomenological Psychology and Aesthetics - CPPA, Department of Psychology, University of Copenhagen.

Carole Tansella, *Section Editor*
